# Exercise capacity after long-term physical activity on prescription provided by physiotherapists

**DOI:** 10.1080/02813432.2025.2450376

**Published:** 2025-01-23

**Authors:** Daniel Karsberg Zotterman, Åsa Cider, Stefan Lundqvist

**Affiliations:** a Unit of Physiotherapy, Department of Health and Rehabilitation, Institute of Neuroscience and Physiology, Sahlgrenska Academy, University of Gothenburg, Gothenburg, Sweden; b Region Västra Götaland, Närhälsan Gibraltar Rehabilitation Centre, Gothenburg, Sweden; c Region Västra Götaland, Department of Occupational Therapy and Physiotherapy, Sahlgrenska University Hospital, Gothenburg, Sweden; d Region Västra Götaland, Centre for Physical Activity, Gothenburg, Sweden; e Region Västra Götaland, Research, Education, Development and Education Primary Health Care, Gothenburg, Sweden

**Keywords:** Physical activity, exercise test, cardiorespiratory fitness, primary health care, physical therapists

## Abstract

**Background:** Research has shown that physical activity on prescription (PAP), used in Swedish healthcare, increases patients’ physical activity, but data are lacking regarding the long-term effects of PAP on exercise capacity. Therefor exercise capacity was evaluated in patients with metabolic risk factors, after 4.5 years of PAP treatment provided by physiotherapists in primary healthcare.

**Method:** This study included 98 patients (49% women; mean age, 56 years) with metabolic risk factors, who were still physically inactive after a previous 6-month PAP treatment. The patients received physiotherapist-provided PAP treatment for 4.5 years, including 11 follow-ups and 6 exercise capacity tests.

**Results:** After 4.5 years, 41 patients completed the final exercise capacity test (58% drop-out rate). Compared to baseline, the whole cohort exhibited a significantly increased exercise capacity (9.1 W, *p* = 0.014) with a small effect size (*r* = 0.27), with no significant differences associated with age or gender.

**Conclusion:** The increased exercise capacity may indicate positive effects on longevity, and consolidates previous findings that long-term behavior change is possible among physically inactive patients. It also demonstrates the feasibility of continuous exercise capacity testing with physiotherapist support in an ordinary primary care setting. The drop-out rate and lack of control group complicate the interpretation of the effects of PAP treatment on the increased exercise capacity. Further research should strive for an RCT study design.

## 1. Introduction

Lack of physical activity (PA) is a global health problem associated with premature death and several non-communicable diseases such as metabolic syndrome, cardiovascular disease (CVD), type 2 diabates and cancer and globally, one-third of adults are estimated to have insufficient PA levels [1]. The World Health Organization (WHO) has highlighted physical inactivity as a leading risk factor for mortality [2]. An increase of PA level can result in a number of health benefits, and regular PA and exercise is important for the prevention and treatment of many different diseases [3].

PA is defined as ‘bodily movement produced by skeletal muscles that results in energy expenditure’ [4], and exercise is distinguished as structured, planned and repetitive PA with the objective to improve or maintain physical fitness [4]. The internationally recommended minimum level of PA is moderate-intensity aerobic PA 150-300 min per week or, alternatively, vigorous-intensity aerobic PA 75–150 min per week, which has been associated with a reduced risk of morbidity and mortality [3].

Cardiorespiratory fitness (CRF), measured as maximum oxygen uptake (VO_2max_) or peak oxygen uptake, is the gold standard measurement for objective evaluation of the cardiorespiratory response to PA and/or exercise [5]. CRF exhibits a strong and inverse relationship to the risk of premature CVD and all-cause mortality [6]. It was stated to be a clinical vital sign by the American Heart Association in 2016, and is recommended to be included in routine clinical healthcare practice [7]. It has been estimated that a CRF increase of 1 mL/min/kg VO_2_ is associated with up to a 9% reduction in the relative risk of all-cause mortality [8, 9]. Among Swedish occupational health controls, a 11% decline in CRF has been measured during the years 1995–2017 [10]. CRF is measured *via* a maximal exercise test that requires extensive personnel and equipment resources, which are typically only found in exercise physiology laboratories. Therefore, for daily clinical practice, a more practical outcome measure is exercise capacity (EC), expressed as power output in watts (W), which can be measured using a symptom-limited cycle ergometer test [7, 11].

Numerous different PA interventions have been shown to improve subjectively and objectively measured PA level among healthy adults [12]. Moreover, in primary healthcare, various PA-promoting interventions have been associated with increases of patients’ PA levels (mainly subjectively measured) [13, 14]. In Swedish healthcare, the physical activity on prescription (PAP) method has been used for this purpose by licensed healthcare professionals over the last 20 years. The Swedish PAP method includes three core elements: patient-centered dialogue; an individually dosed PA recommendation with a written prescription; and individualized follow-up [15, 16]. Interventions using Swedish PAP have exhibited positive effects on self-reported PA, metabolic risk factors, and health-related quality of life [17–22]. Key factors for a successful outcome of PA prescription in general [23], and of the Swedish PAP model in particular, seem to include the individualization of all three core elements, along with continuous follow-up over a long period of time [22, 24–27].

Notably, further research is needed to evaluate the benefits of individualized long-term PAP treatment for different patient subgroups, and for various health outcomes [21, 23, 24, 28, 29]. For example, only three studies have investigated whether PAP can enhance PA as objectively measured using EC [30, 31], or physical capacity measured with a six-minute walking test [32]. Two of these studies [31, 32] showed increased EC and physical capacity after 2 years and 6 months of PAP, respectively, and one study [30] showed unchanged EC after 3 months of PAP. Moreover, few studies have objectively measured PA in older populations [32]. Older adults are reportedly less adherent to PAP [33], and might have difficulties achieving recommended levels of physical activity [34]. Additionally, evaluations of sex-based differences in the benefits of lifestyle interventions on self-reported PA level have produced conflicting results—with some showing that men were more likely to increase their PA level [35, 36], and others indicating a more pronounced effect in women [20].

The present study is part of the Gothenburg PAP study, a five-year primary care intervention targeting adult physically inactive patients with metabolic risk factors where a previous publication showed an increased self-reported PA level in a majority of patients at 6-month follow-up [20]. However, one-third of the patients did not improve sufficiently to be classified as physically active according to the recommended 150–300 min/week of moderate-intensity PA [20]. These non-responders were randomized to either continued PAP treatment at the healthcare center (HCC), or to PT-provided PAP treatment including EC testing [22]. In an initial evaluation of EC among the patients who participated in two years of PT-provided PAP treatment, a significantly increased EC was shown [31]. To our knowledge there is no study that has evaluated EC after PAP treatment lasting longer than two years. In order to support patients’ behavioral change and thereby obtain positive health effects, increased knowledge from long-term follow-up studies on PAP interventions in clinical practice, with evaluations of PA and EC, is needed.

In our present investigation, the primary aim was to evaluate EC after 4.5 years of PT-provided PAP treatment in patients with metabolic risk factors, who were still insufficiently physically active despite a previous 6-month period of PAP treatment at the HCC. The secondary aim was to investigate possible age- and sex-based differences in EC outcomes. We hypothesized an increase in EC among patients with no differences between age and gender.

## 2. Methods

### 2.1. Study design

The present study has a quasi-experimental design, with EC evaluations at baseline and after 4.5 years of PAP treatment. It was conducted as a part of the Gothenburg PAP study, which evaluates the short-term and long-term effects of Swedish PAP. Detailed information regarding the entire study design is available in publications by Lundqvist et al. [20, 22, 37] and Martinsson et al. [31].

### 2.2. Study population

The patient cohort was originally included in the Gothenburg PAP study [20], for which 444 patients were recruited as a convenience sample, between January 2010 to December 2014, from 15 primary HCCs in central and western Gothenburg. The inclusion criteria for participation were the presence of at least one component of MetS (using cut-off values according to the National Cholesterol Education Program (NCEP) classification) [38], being insufficiently physically active (<150 min/week of moderate intensity PA), being willing to receive PAP, and able to understand the Swedish language [20].

**Figure 1.**
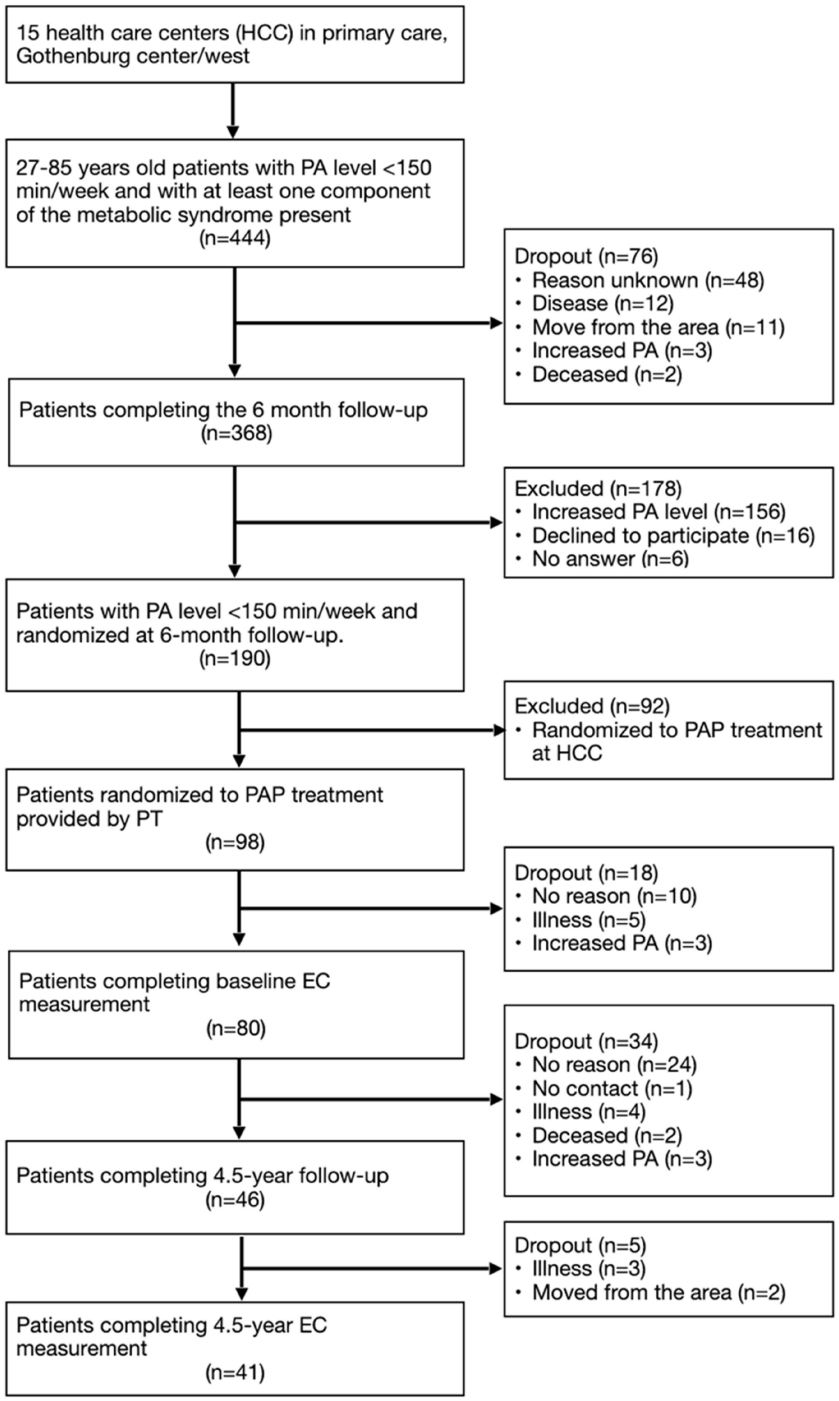
Flow scheme of patients involved in the study.

After 6 months of PAP treatment at the HCC, a majority of patients had improved their PA level significantly [20]. However, 190 patients were still insufficiently physically active and were randomized to either PT-provided PAP treatment including exercise capacity tests (*n* = 98), or continued PAP treatment at the HCC (*n* = 92) [22]. In the present study, we focused on evaluating the 98 patients who were randomized to receive PT-provided PAP (Figure 1).

### 2.3. Intervention

The intervention is described in detail in publications by Lundqvist et al. [20, 22, 37] and Martinsson et al. [31]. Briefly, during a 4.5-year period, the patients received PAP treatment provided by PAP-educated PTs. The PAP treatment included individualized dialogue about PA, an individually dosed PA recommendation with a written prescription, and a fixed follow-up schedule (Figure 1). The patients independently performed the recommended PA, outside the healthcare setting. Each patient was offered a total of 11 follow-up occasions (45-60 min/time) during the 4.5-year period. At each follow-up occasion, the PT had a motivational dialogue with the patient where the physical activity was followed up and possibly supplemented, re-dosed. The new physical activity agreed upon was transferred to a written prescription that the patient received. The EC tests were performed a total of 6 times: at baseline, 4 months, 1 year, 2 years, 3 years, and 4.5 years. The EC test results were discussed with the patients and compared with reference values [39], and thus provided a basis for the continuing motivating dialogue and individually dosed PA recommendations.

In this study, we evaluated data from the measurements at baseline and the 4.5-year follow-up. Data from the cycle ergometer tests were collected by the PTs who conducted the tests and provided the PAP. These PTs were specially educated and trained for this assignment. All other measurement data were collected by the nurses at the HCCs.

### 2.4. Measurements

#### 2.4.1. Anthropometrics

Body mass index (BMI) was calculated from body weight and height. Body weight was measured to the nearest 0.1 kg, with the patient in light clothing without shoes, using an electric scale (Carl Lidén, AFW D300, Jönköping, Sweden). Body height was measured to the nearest 0.5 cm, with the patient in a standing position, without shoes, using a scale fixed to the wall (Personmått PEM 136, Hultafors, Sweden). With the patient in a standing exhaled position, waist circumference (WC) was measured to the nearest 0.5 cm using a measuring tape (Kirchner Wilhelm, Aspberg, Germany) placed on the patient’s skin between the lower rib and the iliac crest. At the HCC, systolic and diastolic blood pressure (BP) were recorded as mean values from 2-3 measurements using a sphygmomanometer (Omron HEM-907, Kyoto, Japan) on the right arm, at the same level as the heart, with the patient in sitting position, after five minutes of rest.

#### 2.4.2. Cycle ergometer test

Exercise capacity (EC) was measured using a standardized symptom-limited cycle ergometer test, following a WHO protocol including a series of stepwise-increased loads [40, 41]. This was considered the most appropriate test for the current patient group and circumstances. The reliability of this specific protocol has been previously evaluated as very good in a study of cardiac patients [42]. At the start of the test, the patient was instructed to cycle with a pedal frequency of 50 rounds per minute (RPM) on a load of 25–50 Watts (W), and to continue for 4.5 min. Then the load was increased by 25–50 W depending on the patient’s capacity, and the patient cycled for 4.5 additional minutes at this higher level. The test was interrupted when the patient reached a perceived exertion of 17 on the Borg rating of perceived exertion (RPE) scale (6-20) [43], if abnormal test results occurred, or if a pedal frequency of 50 RPM was not maintained. Heart rate (HR) and BP (both systolic and diastolic) were measured before the patient started the test and twice within every 4.5-minute interval (BP at 2.5 min and 4.5 min, and HR at 1.5 min and 3.5 min). Borg RPE was estimated at 1.5 min and 3.5 min within each 4.5-minute interval. HR and BP were also measured twice during the recovery phase, up to 4 min after test completion. Additionally, we noted the time and the load when the patient stopped the test, to determine the patient’s highest achieved working rate (W_max_).

The test was performed on a mechanically braked cycle ergometer (Monark Ergomedic 828E, Vansbro, Sweden). Supplementary equipment used for the testing included a blood pressure cuff (AB Henry Eriksson Diagnostik BS-90, Stockholm, Sweden), a stethoscope, a stopwatch, and a heart rate monitor with chest strap (Monark, Vansbro, Sweden).

EC was defined as highest work rate (W_max_) on the cycle ergometer test at 17 on the RPE scale. If the test was interrupted before the patient completed the 4.5-minute exercise period, the highest achieved workload was calculated, as described by Strandell [44], as the heaviest load at which the patient completed 4.5 min of exercise, with the added proportional of the completed time (n) of the next added load (X). The Strandell formula was used as follows:

$$W_{\text{max}}=\left(\text{sub maxial}\;\text{power}\right)+\left(X\cdot n/4.5\right)$$

To exemplify, if a patient completed 4.5 min at 100 W, and then stopped after two minutes at 125 W, the calculation would be as follows:

$$W_{\text{max}}=100+\left(25\cdot 2/4.5\right)=111.1\;W$$

#### 2.4.3. Physical activity level

PA level was estimated using two self-reported PA questions based on a validated questionnaire [45]. The first question asked how many times per week the patient performed 30 min of PA at a moderate intensity level, giving 1 point for each such occasion. The second question asked how many times per week the patient performed 20 min of PA at a vigorous intensity level, giving 1.7 points per occasion. A total score of <5 points indicated an insufficient PA level, corresponding to <150 min/week of moderate intensity, according to the American College of Sports Medicine (ACSM) and the American Heart Association (AHA) [46]. The total score from these two questions was used as an inclusion criterion for participation in the study [20].

### 2.5. Statistical analysis

SPSS version 28.0.1.1 (IBM SPSS Inc., Armonk, NY, USA) was used for statistical analysis. Mean and standard deviation (SD) was presented for normal distributed continuous variables and median and interquartile range (IQR) (Q1–Q3) for non-normal distributed continuous variables. Nominal variables were presented as absolute and relative numbers. No sample size calculations were made as the cohort size for the present study was predetermined as it was part of a larger cohort in the Gothenburg PAP study where the patients had previously been randomized to PAP treatment *via* PT’s. Differences in PA level, between baseline and the 4.5-year follow-up, were analyzed using the paired sample t-test. Wilcoxon signed-ranks test was used to analyze differences in EC between baseline and the 4.5-year follow-up. Mann-Whitney U test was used to analyze differences in EC between female versus male subgroups, and between the dichotomized age groups <58 years old versus ≥58 years old. Age dichotomization was conducted to enable comparison of two groups of similar size, and was based on the median age for the whole cohort. An independent-samples *t-*test or Mann–Whitney U-test was used to perform subgroup analyses, comparing baseline characteristics between the follow-up group versus the drop-out group, and between females versus males, based on the data level. The alpha level that determined statistical significance was set to <0.05. Effect size (ES) was calculated as r = z/√n [47] (r = ES, z = Wilcoxon test value, n = total number of pairs), where 0.1 indicated a small ES, 0.3 a medium ES, and 0.5 a large ES [48].

### 2.6. Ethical considerations

The Gothenburg PAP studies [20, 22, 31, 37] received ethical approval from the Regional Ethical Review Board in Gothenburg, Sweden (Dnr 529-09). Participation was voluntary, and patients received written and verbal study information before they gave their written consent. The patients could interrupt their participation, and have their collected data removed at any time. The risks of adverse events for sedentary individuals starting PA were acknowledged [49] and considered.

## 3. Results

A total of 46 patients (47%) attended the 4.5-year follow-up, of whom 41 patients (42%) performed the final exercise capacity test. Figure 1 presents the flow of study participants.

### 3.1. Baseline characteristics

The whole group had a mean age of 56 years, and comprised an equal distribution of men and women. At the group level, the patients were overweight and had a waist circumference above the recommended limit. Hypertension was present in 77%, hyperlipidemia in 52%, and 36.5% had hyperglycemia. The patients were insufficiently physically active at a level corresponding to 75 min of moderate intensity PA per week (Table 1). We found no statistically significant differences at baseline between the follow-up group and the drop-out group, except for a higher WC in the drop-out group. Comparing baseline characteristics between men and women revealed that the women were significantly older, and had a lower waist circumference.

**Table 1.** Baseline characteristics of the included patients.

Variable (*n*)	Value
Age, years (98)^a^	56.4 (10.2)
Sex (98)^b^	
-Male	50 (51)
-Female	48 (49)
PA level, activity points (98)^a^	2.4 (1.4)
BMI, kg/m2 (96)^a^	32.3 (5.6)
Waist circumference, cm (98)^a^	108.2 (14.3)
BP Systolic, mmHg (98)^a^	132.7 (17.1)
BP Diastolic mmHg (98)^a^	82.2 (10.0)

Data are given as ^a^mean (standard deviation) or as ^b^number (percentage). PA, physical activity; BMI, body mass index; BP, blood pressure.

### 3.2. Outcomes

Of the 46 patients who completed the 4.5-year follow-up, 34 patients (74%) had increased their PA level by an average of 5.7 points compared to 2.3 points at baseline (*p* < 0.001), according to the PA questions and 24 patients (52%) had improved their PA from inadequate to sufficient, according to the public health recommendations of the ACSM/AHA [46]. The most commonly prescribed physical activity was walking and/or gym training (73%), 30-60 min (95%) on a moderate intensity level (86%), 2-6 days per week (71%).

At the 4.5-year follow-up, the whole group showed a statistically significant improvement in EC (average of 9.1 W; *p* = 0.014), with a small effect size (*r* = 0.272) (Table 2). Subgroup analyses based on gender and age revealed similar improvements of EC values as observed in the whole group, but these improvements in subgroups were not statistically significant (Table 2).

**Table 2.** Difference in exercise capacity (ΔW) between baseline and 4-5-year follow-up for the whole group and the subgroups based on gender and age.

Group (n)	Baseline^a^, Watts	4.5-years^a^, Watts	ΔW^b^	*P*-value^c^	ES
Total (41)	91.5 (36.1)	100.6 (33.5)	9.1 (9.9)	0.014	0.272
Females (24)	77.8 (33.7)	87.3 (31.0)	9.6 (12.2)	0.077	
Males (17)	110.8 (30.8)	119.3 (28.2)	8.5 (7.7)	0.079	
≥58 years (20)	87.7 (36.7)	93.0 (35.1)	5.3 (6.0)	0.121	
<58 years (21)	95.0 (36.0)	107.8 (31.0)	12.8 (13.5)	0.051	

Data are given as ^a^mean (standard deviation) or as ^b^mean (%). *P*-value was determined by ^c^a Wilcoxon signed ranks test. Statistical significance was set at *p* ≤ 0.05. Δ, delta; W, Watts; ES, effect size.

We found no statistically significant differences in the change of EC between men and women, or between patients who were ≥58 years old versus <58 years old (Table 3).

**Table 3.** Difference in exercise capacity (ΔW) between baseline and 4.5-year follow-up based on males versus females and <58 years versus ≥58 years.

Subgroup (*n*)	ΔW^a^	P-value ^b^
Males (17)	8.5 (19.6)	0.905
Females (24)	9.6 (22.1)	
≥58 years (20)	5.3 (14.5)	0.426
<58 years (21)	12.8 (25.3)	

Data are given as ^a^mean (standard deviation). *P*-value was determined by ^b^a Mann Whitney U test. Statistical significance was set at *p* ≤ 0.05. Δ, delta; W, Watts.

## 4. Discussion

This study included patients with metabolic risk factors, who had insufficient PA levels after a previous 6-month period of PAP treatment, and who subsequently completed a 4.5-year period of PT-provided PAP. The primary outcome was an increased EC in this patient group. The EC outcomes did not differ between men versus women, or between older versus younger patients. The 4.5-year results showed sustainment of the previously reported improvement of EC in the 2-year follow-up study by Martinsson et al. [31]. A majority of the patients also reported an increased PA level, with half of patients reaching an adequate PA level according to the public health recommendations.

The present results have clinical relevance, as they demonstrate the possibility to achieve long-term sustained PA-related behavioral change in physically inactive patients who are presumably low-motivated, as implied by their nonresponse to the previous 6-month PAP treatment [20]. Since physical inactivity is a leading global cause of morbidity and mortality [2], and CRF has been reported as declining in Sweden [10], this is a priority global and national issue for healthcare in general, and particularly for the PT profession.

Numerous recommendations have called out the need for and importance of measuring patients’ CRF for risk classification and for healthcare management [7, 50, 51], due to its strong and inverse relationship to the risks of premature CVD and all-cause mortality [6, 8, 9]. Besides its main purpose, the present study also demonstrates that it is feasible and manageable to conduct these measurements and follow-ups for patients in a primary care setting, over many years, at least in a publicly funded health care system similar to the Swedish one.

The risk reduction associated with an increased CRF varies in different studies, with different study populations. Laukkanen et al. [8] examined 579 men, aged 42–60 years, and estimated that all-cause mortality risk was reduced by 9% per every 1 mL/min/kg higher VO_2max_. Ekblom-Bak et al. [9] studied a population of 266,109 people from an occupational health service screening (18–74 years old, 47% women), and found risk decreases of 2.3% for all-cause mortality, and 2.6% for CVD mortality, per VO_2max_ increase of 1 mL/min/kg. Notably, a 9.1% reduction of all-cause mortality risk was observed among persons in the lowest estimated VO_2max_ category. Since there is a compatible relationship between CRF and EC [52], the EC values in our present study could be viewed in relationship to previous studies by examining the percent change. Our current study population had a mean EC value of about 100 W (Table 2), which can be translated into a CRF value of 21 mL/min/kg for a person weighing 70 kg [53]. Thus, the increased EC of 9.9% corresponds to CRF increase from 21 to 23 mL/min/kg, i.e. an improvement of 2 mL/min/kg. Compared to the aforementioned reductions of mortality risk (2.3% and 2.6%), this would be equivalent to risk reductions of 4.6% for all-cause mortality, and 5.2% for CVD mortality. Assuming that the patients are in the least-fit group, which is highly likely, the 9% reduction in all-cause mortality risk per gained 1 mL/min/kg is more valid, and would translate to an 18% reduction in all-cause mortality risk. Furthermore, when examined relative to the 4.5-year duration of this study, the increase of EC is potentially even more relevant, as CRF typically declines with age [54].

Compared to the previously published 2-year follow-up study [31], the average EC improvement (ΔW) was similar, and even somewhat higher, after 4.5 years (9.1 W vs. 7.6 W). This can be seen as a very promising result, since other comparable studies examining long-term effects of PA-interventions in sedentary patients have shown decreased physical fitness from year 2 to year 4 [55]. It is possible that the positive long-term outcome in the present study can be explained by the individually customized and skillfully supported PAP treatment—factors that have been emphasized as crucial by patients in other studies [25–27]. At the 4.5-year follow-up, all subgroup analyses showed improvements in EC, but did not reach statistical significance, although the <58 years group was close with a *p*-value of 0.051. In contrast, at the 2-year follow-up, significant improvements were observed in 3 out of 4 subgroups. Presumably, this difference is explained by the decreased subgroup sizes at the 4.5-year follow-up. Additionally, the ES was a little lower at the 4.5-year follow-up compared to at the 2-year follow-up, which is likely primarily due to larger standard deviations. The ES may have also been affected by the content and the volume of the performed PA, which exhibit a crude dose-response relationship to CRF [56]. Since these differences have not been analyzed, it is difficult to draw specific conclusions about the cause of the differences between the 2-year and the 4.5-year follow-up. However, a relatively small magnitude of ES could be expected in a long-term real-life study involving PA behavior, since PA measurement is complex and affected by many factors at the individual, social, psychological, behavioral, and environmental levels [57].

The drop-out rate was 58% at the 4.5-year EC follow-up, compared to 51% at the 2-year follow-up, which represents 7 fewer patients in absolute numbers. Other PAP studies have reported drop-out rates of between 30–38% at 6–12 months of follow-up [17, 32, 58], and 41–48% at 2 years of follow-up [59, 60]. A 5-year follow-up study examining EC in older males with chronic obstructive pulmonary disease reported a drop-out rate of 50% [61]. The Swedish Council on Technology Assessment in Health Care (SBU) has stated that there is a need for long-term evaluations of PA interventions, and has implied acceptance of higher drop-out rates in studies with particularly long follow-up [62]. Notably, a substantial number of the patients in this study dropped out before the first EC test, at baseline, and just before the last test. The majority of the patients who dropped out gave no stated reason for their absence. The ergometer cycle test is associated with some physical effort and time consumption, which could likely be reasons for a higher drop-out rate compared to in studies with more convenient non-physical follow-ups.

For measuring EC, a symptom-limited cycle ergometer test has the benefits of being a safe and adjustable test, which have been highlighted as factors of priority when performing exercise evaluations on patients [63]. The EC test is more easily applied and less expensive than measuring CRF *via* maximal tests, which require, e.g. more advanced equipment.

Since the cycle ergometer measurements were conducted in the present study, other sub-maximal tests have been evolved. In future evaluations, other tests could be considered, to facilitate more precise and comparable estimations. Some caution must also be taken when interpreting the outcome of a cycle test, since the relationship between work rate and VO_2_ is not entirely accurate, especially at higher work rates [64]. It must also be acknowledged that lower extremity strength can sometimes affect the results of symptom-limited cycle tests [65].

### 4.1. Limitations

The study has several limitations, such that the results must be interpreted with caution. There was no control group; therefore, it is not possible to draw specific conclusions about the effects of the PAP intervention on the increased EC. However, previous PAP studies with control groups [18, 22, 66] and without control groups [17, 67] have shown positive effects on self-reported PA, which is a prerequisite for an improvement in EC. Notably, the superiority of an RCT design has been debated [68], and it has been suggested that RCTs are often supplemented with data from observational studies including representative real-world patient samples [69].

The present study did not analyze the content or volume of the performed PA, and its possible relationship to the EC change, or whether any other baseline characteristics (apart from gender and age) were correlated with better outcome. Moreover, this study did not analyze when during the 4.5 years the EC change occurred. These aspects could be topics for further evaluations of the present study material, to better understand why some patients get better results, and how the intervention can be optimized.

Furthermore, the cohort size of the present study was predetermined as it was part of a larger cohort in the Gothenburg PAP study [20] where the patients had previously been randomized to PAP treatment *via* PTs. The lack of sample size calculations and the high drop-out rate, may affect the external validity, especially for the subgroup results. Notably, a drop-out rate of this magnitude is probably to be expected, given the context and timeframe of the present study. Another methodological limitation was that the PT who conducted the EC tests also provided the PAP treatment for the patient. This could have been avoided by having different PTs providing the PAP and EC tests. However, this study was a survey of daily clinical practice, with no extra resources to employ additional PTs.

## 5. Conclusions

To our knowledge, this is the first study to evaluate EC after a 4.5-year PT-provided PAP intervention in physically inactive patients with metabolic risk factors. The results showed an increased EC of 9.1 W for the whole cohort who fulfilled the treatment, with no subgroup differences based on age or gender, indicating positive effects on longevity. The improvement in EC is clinically relevant as it consolidates previous PAP study findings of the possibility of long-term PA behavior change among physically inactive patients. The study also demonstrates the feasibility of continuous EC testing with PT support in an ordinary primary care setting. The drop-out rate and the lack of a control group complicate the interpretation of the effects of PAP treatment on the increased EC. Future research should strive for randomized controlled trials in which sufficient cohort sizes can be estimated based on the drop-out rates in this study. Further analyses of the present study material may also be of interest, especially concerning the content of the PA, and its relation to the EC change.

## Data Availability

The data presented in this study are available on request from the corresponding author.
